# Mouthrinses against SARS-CoV-2: anti-inflammatory effectivity and a clinical pilot study

**DOI:** 10.1007/s00405-021-06873-8

**Published:** 2021-05-22

**Authors:** Matthias Schürmann, Mohamed Aljubeh, Carsten Tiemann, Holger Sudhoff

**Affiliations:** 1 Department of Otorhinolaryngology, Head and Neck Surgery, Medical Faculty OWL, Bielefeld University, Campus Klinikum Bielefeld, Teutoburger Str. 50, 33604 Bielefeld, Germany; 2 Labor Krone, Laboratory for Medical Diagnostics, Bad Salzuflen, Germany

**Keywords:** Covid-19, Mouth wash, Anti-inflammatory, Zinc, Dexpanthenol

## Abstract

**Purpose:**

The scope of this research endeavor was the determination of the applicability of over the counter mouthwash solutions in reducing the viral load in the saliva of COVID-19 patients and hence decreasing their infectivity. Beyond that, new experimental mouthwashes were investigated in terms of a possible positive immune modulation, which might offer an additional opportunity for a positive pharmaceutical effect.

**Methods:**

The effectivity of the mouth washing solution was determined on 34 hospitalized COVID-19 patients by measuring the viral load by RT-qPCR in pharyngeal swabs, which were taken before and after rinsing. The inflammatory modulation thru the experimental solutions was assayed in an in vitro model of virus infected nasopharyngeal epithelium cells.

**Results:**

The clinical pilot study demonstrated that the mouth rinsing solution was able to reduce the viral load by about 90% in the saliva of most patients. This reduction was determined to persist for about 6 h. In the experimental solutions, the ingredients dexpanthenol and zinc were able to reduce the expression of proinflammatory cytokines in the cell culture model, while the antiviral response was not altered significantly.

**Conclusion:**

We recommend the application of mouth wash solutions to COVID-19 patients, since our results indicate a reduction in infectivity and might govern the protection of health care professionals. Further improvement to the over the counter formulation can be made by utilizing zinc and dexpanthenol, as they which might be beneficial for the patients’ health.

**Supplementary Information:**

The online version contains supplementary material available at 10.1007/s00405-021-06873-8.

## Introduction

The severe acute respiratory syndrome coronavirus 2 (SARS-CoV-2) as the cause of coronavirus infectious disease 2019 (COVID-19). COVID-19 has become a global source of morbidity, mortality and social disruption since its emergence in Asia in late 2019 and subsequent pandemic spread. Despite intense efforts including vaccination campaigns, the virus pandemic continues requiring further preventive measures. Due to the airborne transmission, the use of antiseptic mouth rinses against SARS-CoV-2 seems an obvious possibility to reduce the viral load and thereby decrease the likeliness of viral transmission. Mouthwashes are widely used solutions, to reduce the number of micro-organisms in the oral cavity and colony-forming units in aerosols [7]. Since SARS-CoV-2 enters cell via the ACE-2 receptor and ACE-2 is highly expressed in salivary glands and oral cavity epithelial cells [6, 31], SARS-CoV-2 shows a high reproduction rate in oral tissues. Hence it is suspected that the saliva in the oral cavity may act as a potential reservoir for the transmission of the highly contagious novel coronavirus. In accordance with that, SARS CoV-2 has been detected in high amounts in the saliva of the COVID-19 patients [28]. Further studies identified saliva as a source of viral spread [17], mainly because viruses can be transmitted through aerosol generation from the oral cavity through increased pressure due to coughing, sneezing or speaking (reviewed in [16]. Hence commonly applied over-the-counter mouth rinses could be an easy application to reduce the overall infectivity of COVID-19-positive patients. Numerous investigations were already undertaken to reduce the viral load in COVID-19 patients by gargling with mouth rinsing solution. Several over‐the‐counter mouthwash products showed promising results in in-vitro experiments [18, 19]. Until recently, only povidone iodine was able to show promising results in clinical practice and was investigated in controlled clinical trials utilizing larger study populations [3]. The larger population (*n* > 1000) made it possible to detect positive changes in the clinical burden. The authors even suggested a decrease in mortality upon application of this strong virucidal agent. Unfortunately, side effects, e.g. disturbance of epithelial barrier [24] or thyroid dysfunction occurred in patients exposed to long-term povidone iodine gargling [8]. Notably, a minor viral clearance of the throat was already achieved by physical means via gargling with tap water [20]. This effect might be enhanced by utilizing surfactant based cleaning products like baby shampoo known to inactivate SARS‐CoV‐2 in vitro [11, 19]. The reduction of viral load via the mouth washing procedures may be affected by ingredients already used for oral health care products.

It is known that many species of viruses, including SARS-CoV-2, have evolved mechanisms to dampen the antiviral effect of inflammation caused by interferon-beta (IFN-β), MX-1 or OAS-1 [5], but simultaneously enhancing the inflammatory signaling (via IL-6 and various CXCLs). This will promote the fatal inflammatory state of the infected tissue [1, 9]. Particularly, GM-CSF was reported to be higher in critically ill patients [29] and high expression of CXCL-9, CXCL‐10 and interleukin‐6 (IL‐6) resulting in a poor prognosis in SARS‐CoV-2 infections [2, 13, 27]. Only the glucocorticoid dexamethasone provides a unique anti-inflammatory effect able to provide control against a cytokine storm and improves clinical outcome [22].

In this combined in vitro and in vivo study, we investigated the clinical action of an over the counter mouth wash and tried to find starting points for an optimized formulation of an experimental mouth rinse formulations based on in vitro experiments. Our clinical data determined the reduction of the viral load of SARS-CoV-2 infected hospitalized patients upon application of an over-the-counter product. Furthermore, the in vitro experiments with anti-inflammatory agents known for insignificant side effects were conducted. One was dexpanthenol, which is known to reduce inflammation and IL-6 release in respiratory epithelium [15] and zinc known to decreases NF-κB activation resulting in decreased expression of proinflammatory target genes [12]. By this, we intended to restore a proper inflammatory response in epithelial cells which is thrown out of balance by a viral infection.

## Materials and methods

### Cell culture

For culture of nasopharyngeal epithelium tissue extracted during head and neck surgery was utilized. Immediately after removal the tissue was stored on ice and transported to the cell culture lab. After removing excess connective tissue and clotted blot the mucosa was chopped into small pieces (approx. 2mm^3^) and digested with collagenase (0.375 U/ml in PBS, NB4; SERVA Electrophoresis GmbH, Germany) at 37 °C for 2 h. If necessary, erythrocyte lysis buffer (155 mM NH_4_Cl, 10 mM KHCO_3_, 0,1 mM EDTA at pH 7.3) was applied after centrifugation of the suspension and decantation of the collagenase. Subsequently, the pelleted cells were resuspended in PneumaCult™-Ex Plus media (STEMCELL Technologies Inc., Canada) and cultivated for 3–7 days in T25 cell culture flask (Sarstedt, Germany). During this time the media was changed every second day. Following this pre-cultivation, the cells were detached with Accutase (Capricorn, Germany), seeded in the designated multiwall plates (Starlab, Germany) and further processed for the experiments described below.

### TLR-3 stimulation and screening of inflammatory mediation by test substances

To simulate viral infection cells were seeded in PneumaCult™-Ex Plus media with a density of 10^4^ cells per cm^2^ in six well plates. After 24 h the cells were incubated with different concentrations of the TLR-3 agonists Poly (I:C) or 10 µg/ml Poly (I:C) in combination with 1% (v/v) of the test substances A–F or 5% of the test substances E*, F* and D. After 4 h of incubation at 37 °C with 5% CO_2_ the cells were further processed for RNA isolation and RT-qPCR.

### RT-qPCR

The cells were washed two times with PBS and the mRNA was derived from the homogenized cells suspension by a RNA extraction kit (innuPREP DNA/RNA Mini Kit 2.0; Analytik Jena, Germany) and transcribed to cDNA (RevertAid First Strand cDNA Synthesis Kit, Thermo Fisher, USA). RT-qPCR was performed using the magnetic induction cycler (MIC, BMS, Australia) utilizing a ready to use master mix (Luna Universal qPCR Master Mix; NEB, USA) containing 200 nM Primer (Table 1) in a 10 µl sample volume as technical triplicate. As reference gene for quantification served the GAPDH transcript.

**Table 1:** Primers utilized in this study

TNF fw/rev	AAG CCC TGG TAT GAG CCC ATC TAT	AGG GCA ATG ATC CCA AAG TAG ACC
IL6 fw/rev	GCAAAGAGGCACTGGCAGAAAACA	TTCTGCAGGAACTGGATCAGGACT
CXCL-9 fw/rev	CCTGCATCAGCACCAACCAA	TTTTCTCGCAGGAAGGGCTTG
CXCL-10 fw/rev	CCTTTCCCATCTTCCAAGGGT	GGAGGATGGCAGTGGAAGTC
GMCSF fw/rev	TCATCTGGCCGGTCTCACTC	TCATCTGGCCGGTCTCACTC
IFNB fw/rev	GACGCCGCATTGACCATCTA	TCTCATTCCAGCCAGTGCTA
OAS1 fw/rev	CCTGGTTGTCTTCCTCAGTCC	CTGGACCTCAAACTTCACGGA
MX1 fw/rev	AAGATGGTTGTTTCCGAAGTGG	TCAGTAATAGAGGGTGGGATGC

### Clinical pilot study

The clinical pilot study was conducted in the Department of Otolaryngology, Head and Neck Surgery, Campus Klinikum Bielefeld, Bielefeld University. 34 SARS-CoV-2 positive hospitalized patients were recruited for an observational study approved by the ethical committee of Ruhr-Universität Bochum (Bad Oeynhausen, Az 2020-726_1) and gave their informed consent prior to their inclusion in the study which was conducted in accordance with the ethical standards laid down in the Declaration of Helsinki. The patients gargled the mouthwash (Linola sept, Dr. August Wolff GmbH & Co KG, Bielefeld/Germany) for 1 min. Directly before and 5 min after gargling pharyngeal swabs using a standardized protocol were taken and sent for SARS-CoV-2 analysis (Labor Krone, Bad Salzuflen/Germany). To investigate the time course of viral load development after gargling, additional pharyngeal swabs were taken from five patients after 2 h, 4 h and 6 h. Real-time polymerase chain reaction (RT-qPCR) for the S-, N- and R-genes with cycle threshold (Ct) for SARS-CoV-2 was performed (Seegene, Seoul, Korea, according to the suppliers instructions). For the analysis the most sensitive S-gene Ct-values (pre and post rinsing) descriptive statistics were used. In case of missing Ct-values pre rinsing, data were excluded from the descriptive statistics. Missing Ct-values post rinsing (no virus detected) were replaced with a Ct-value of 37 for statistical reasons. Ct of 37 indicates a minimal existing viral load. The viral load was derived from the Ct value with the aid of a regression curve obtained from a solution containing a standardized viral load. The viral loads of the patients obtained in this way (before and after rinsing and over the following hours) are used to calculate the reduction in viral load and the relative reduction of viral load for each patient.

To investigate the temporal development of the pharyngeal viral load patients we sampled the viral load of five patients at different time points (0 h, 2 h, 4 h and 6 h) after gargling.

## Results

### Screening for interesting formulation of mouth wash and establishment of cell culture model to investigate the modulation of inflammation

Different test substances were supplied to the laboratory (Table 2).

**Table 2:** The formulations of the different test substances investigated regarding their inflammatory modulation

Name	Product/composition
A	Biorepair^®^ Zahnmilch: aqua, sorbitol, xylitol, zinc hydroxyapatite, cellulose gum, zinc pca, aroma, peg-40, hydrogenated castor oil, sodium lauryl sulfate, sodium myristoyl sarcosinate, sodium methyl, cocoyl taurate, lactoferrin, sodium hyaluronate, sodium saccharin, sodium benzoate, phenoxyethanol, benzyl alcohol
B	Karex^®^ Mouthwash: aqua, glycerin, hydroxyapatite, xylitol, aroma, disodium pyrophosphate, tetrapotassium, pyrophosphate, zinc pca, hydroxyethylcellulose, propylene glycol, sodium methyl cocoyl, taurate, sodium hydroxide, aloe barbadensis leaf juice powder, chamomilla recutita flower, extract, cetylpyridinium chloride, potassium acesulfame
C	Second Source Biorepair^®^: aqua, glycerin, zinc hydroxyapatite, xylitol, cellulose gum, microcrystalline cellulose, aroma, peg-40 hydrogenated castor oil, hydroxyethylcellulose, zinc pca, allantoin, sodium methyl, cocoyl taurate, sodium myristoyl sarcosinate, propylene glycol, sodium hyaluronate, sodium, saccharin, sodium hydroxide, sodium chloride, phenoxyethanol, benzyl alcohol
D	Aqua, carrageenan, zinc acetate and zinc PCA (pyrrolidone-carboxylate), dexpanthenol, sodium hyaluronate, sodium benzoate, citric acid
E	Aqua, carrageenan, zinc acetate and zinc PCA (pyrrolidone-carboxylate), dexpanthenol, sodium hyaluronate, phenoxyethanol, citric acid
F	Aqua, carrageenan, zinc PCA (pyrrolidone-carboxylate), phenoxyethanol
E*	Aqua, zinc, dexpanthenol
F*	Aqua, zinc
D	Aqua, dexpanthenol

**Fig. 1:**
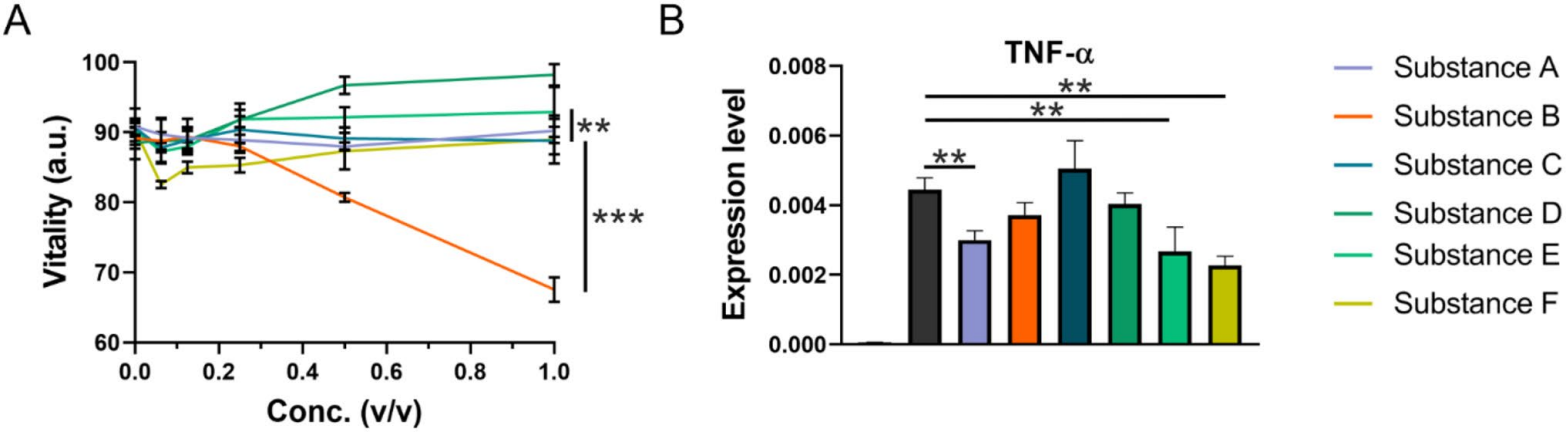
Vitality of oral epithelial cells after incubation with the six test substances. **a** The cells were incubated for 24 h with different v/v ratios of the test substances. The vitality was measured in terms of metabolic activity via an MTS assay. The test substance A, C and F exhibited only minor effects on the metabolic activity. The test substance D and E showed a positive effect on the vitality of epithelial cell. The test substance B decreased the metabolic activity of the assayed cells. (one tailed Mann–Whitney-*U* with 95% confidence interval, ** ≤ 0.01, *** ≤ 0.001) **b** The cell culture model of epithelium treated with 10 µg/ml Poly (I:C) showed a reproducible strong upregulation of inflammatory marker TNF-α. Interestingly, it also showed a significant downregulation of this inflammation upon treatment with 1% (v/v) of test substance A, E and F. (Depicted: mean and standard deviation; *t* test with welch correction, two tailed with 95% confidence interval, ** ≤ 0.01)

Test substance A–B are commercially available product whereas already established as a mouth wash the all the other substances were experimental formulations. First, all test substances (Table 2) were assayed for in vitro biocompatibility via an MTS assay and their anti-inflammatory properties (Fig. 1a). The test substance A and C exhibited no effect on the vitality. The test substance D showed a positive effect on the vitality of epithelial cell, with a significant elevation of 10% above the ground level at 0.5% and 1% v/v. In contrast to that, the test substance B decreased the metabolic activity of the assayed cells. This decrease was dose dependent and can be estimated to lay around 10% at 0.5% v/v and around 20% at 1% v/v. The test substance E showed only a slight but not significant elevation above the ground level. The test substance F showed a significant decrease around 5% at low concentrations (0.0625% and 0125% v/v). This was balanced out at higher concentration. Hence all substances except of B demonstrated a fair in vitro biocompatibility.

Subsequently, we assayed all test substances according to their anti-inflammatory properties. In order to optimize the simulation of a viral infection in our epithelial cell populations in vitro, Poly (I:C) was administered with 1 µg/ml, 3 µg/ml and 10 μg/ml to determine the optimal dose, to measure inflammatory and immune-modulatory cell signaling pathways in primary cell cultures and find the most sensitive inflammatory markers to measure the pharmaceutical effect of the test substances. TNF-α showed a similar and robust upregulation already at 1 µg/ml and can be used as marker for TLR-3 induced inflammation. At a concentration of 10 µg/ml Poly (I:C) a stable and strong upregulation of all key inflammatory mediators was observed (Fig. S1). Therefore, we supplemented 10 µg/ml Poly (I:C) and 1% (v/v) of the test substances into the medium for the investigation of inflammatory regulation through the different substances (Fig. 1b). Only E and F showed the strongest downregulation of the TNF-α expression initially induced by TLR-3 stimulation (1.8-fold and 2.0-fold, respectively).

### Applicability in cell culture of vitality of test substances E and F and improvement for in vitro testing

Test substances E and F were the most promising candidates for further anti-inflammatory investigations. Beyond this the substances E and F were the only ones with a simplified composition which allowed us to investigate the anti-inflammatory potential of dexpanthenol and zinc in our cell culture model. To gain a higher in vitro concentration of these substances, without further dilution of the cell culture medium, we investigated differently concentrated formulations of these recipes.

Unfortunately, the test substances E100 and F100 showed the decency to coagulate and precipitated components of the cell culture medium (Fig. S2a). In contrast to that much less coagulation even at high concentrations of 3% v/v were observed in E10 and F10 formulations. Since we needed to test if that amount of coagulation is negligible, we compared the viability in titration of E and E10 or F and F10, respectively (Fig. S2b). Results demonstrated that test substances E and E10 obtained similar outcomes compared to F and F10 and are likewise suitable for further in vitro testing. Since we could detect unknown coagulum (cf. Fig. S2a), we decided to keep the original concentration of the test substance for further in vitro testing.

**Fig. 2:**
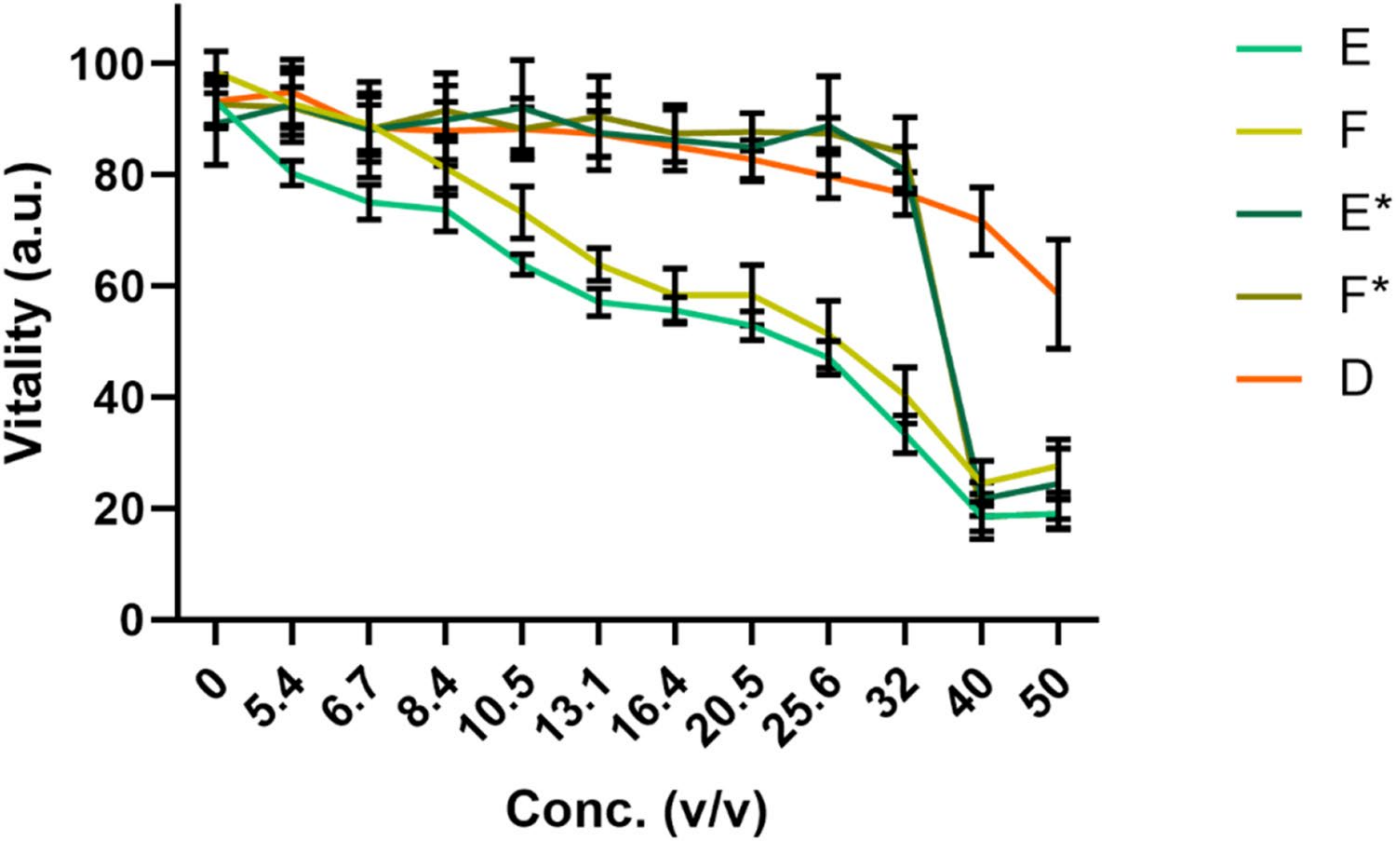
Viability of oral epithelial cells treated with the four test substances. The exclusion of Carrageenan and phenoxyethanol resulted in a highly in vitro biocompatible formulation allowing high concentration of E*, F* and D to be used in vitro without causing severe cellular stress. Isolated from treated and untreated cells. RT-qPCR results are shown in Fig. 3

Subsequently, we investigated formulation E and F, which were initially optimized as mouth gargle with regard to improved suitability for in vitro culturing. To reduce intracellular stress, which might alter the inflammatory response of the culture, we removed the Carrageenan, utilized for its gelling, thickening, and stabilizing properties, and the preservative phenoxyethanol from the formulations E and F and termed them E* and F*. Since E contained Zinc and dexpanthenol as biological active ingredient and F only contained zinc, we introduced a further test substance containing the same concentration of dexpanthenol as in E and termed this substance D. We again tested this new test substance for their biocompatibility in an in vitro test (Fig. 2). We found that this improved the in vitro biocompatibility notably. Therefore, we decided to conduct the final assessment of the inflammatory mediation by the test substance with the new formulations E*, F* and D, which were optimized for in vitro testing allowed high concentrations of up to 30% (v/v) (For E* and F*) and 40% for D without significant reduction in vitality or induction of cellular stress, respectively).

**Fig. 3:**
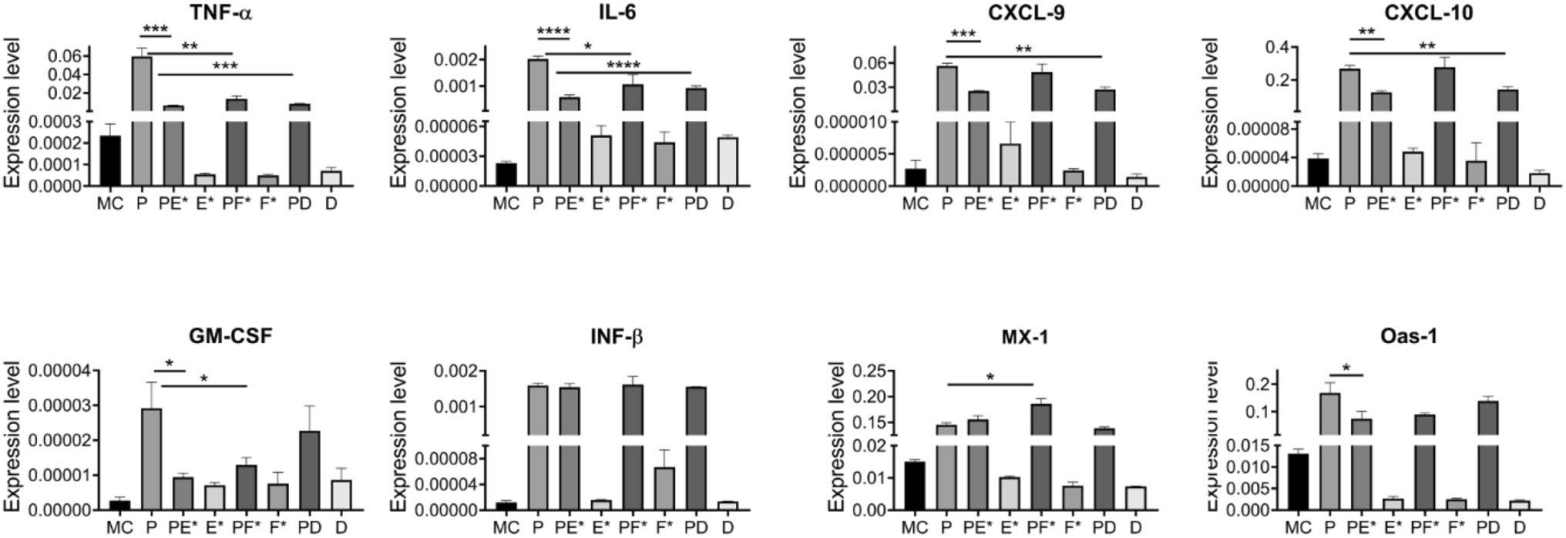
Investigation of action of the test substances on cells treated with the test substances E*, F* and D. Compared to the medium control (MC) a strong upregulation of all transcripts was observed upon application of Poly (I:C) (P). More importantly, the expression of the cytokines (TNF-α, IL-6, GM-CSF) and chemokines (CXCL-9 and CXCL-10) was significantly downregulated by additional application of test substance E*. Some were more sensitive to dexpanthenol (chemokines) and some susceptible to zinc (GM-CSF). The antiviral transcripts (INF-β, MX-1 and OAS-1) on the other side were slightly upregulated (MX-1 by F*), slightly downregulated (OAS-1 by zinc + dexpanthenol) or not influenced at all (INF-β) (*t* test with welch correction, two tailed with 95% confidence interval,* ≤ 0.05, ** ≤ 0.01, *** ≤ 0.001, **** ≤ 0.0001). Three anionic surfactants led to a significant reduction of the viral load in the pharynx

### Assay for modulation of inflammation by the test substances

In terms of biocompatibility of the test substance (Fig. 2) we applied 5% v/v of the test substance and 10 μg/ml Poly (I:C) to the cell culture model. After 4 h of incubation total RNA was determined.

The new formulations of the mouth washes enabled the investigation of the inflammatory modulation by the two active ingredients zinc and dexpanthenol in our cell culture model of virus-infected epithelial cells. We found that the significant 250-fold upregulation of the major cytokines TNF-α was damped by a factor of about 4.4 with zinc alone and around 7.0 with dexpanthenol (with or without zinc). The other central cytokine of SARS-CoV-2 driven cytokine storm IL-6 was also strongly upregulated (90-fold); again zinc and dexpanthenol counteracted this trend and reduced this upregulation by a factor around 2, when administered alone, and a factor of about 3.5 in combination. All these anti-inflammatory actions were significant with *p* values between 0.03 and smaller as 0.0001. The two cytokines CXCL-9 and CXCL-10 were heavily upregulated (21.000-fold and 7.000-fold, respectively) and did not react to a treatment with zinc. But both chemokines were reduced by a factor of about 2 independent from a combinatory treatment of dexpanthenol with zinc or with dexpanthenol alone in a highly significant manner (with *p* values between 0.003 and 0.004). In case of GM-CSF this effect was the other way around and the downregulation by a factor of about 2.5 was achieved with zinc alone as well as a combination of dexpanthenol and zinc while dexpanthenol had no effect at all (both *p* values around 0.04). Most interestingly, the strong upregulation of the antiviral target INF-β, MX-1 and OAS-1 was not distinctively downregulated by the treatment with zinc or dexpanthenol. In detail, the strong upregulation (130-fold) of INF-β was not influenced, neither by zinc nor by dexpanthenol or the combinatory treatment. The expression of the antiviral MX-1 was even slightly further increased via the treatment with zinc (1.2-fold). Only the antiviral target OAS-1 exhibited a small (2.3-fold) downregulation after application of the combinatory treatment.

### Clinical pilot study

34 SARS-CoV-2 positive hospitalized patients were recruited for an observational study. We utilized the over-the-counter product Linolasept^®^ mouthwash with an analogous composition to Biorepair^®^ (Test substance A). The selection of this product was based on its availability as an established mouth gargle. Five datasets were excluded due to missing Ct-values of the pre-rinse-samples. The overall mean of Ct-values before rinsing was 26.0 (standard deviation 5.8) and a median of 28.0. The overall mean of Ct-values after rinsing was 29.1 (standard deviation 6.1) and a median of 31.0 (Fig. 1). The mean values show an increase of the Ct-values of 3.1 (standard deviation 3.6). This indicates a reduction of the viral load in the pharynx of about 90%.

**Fig. 4:**
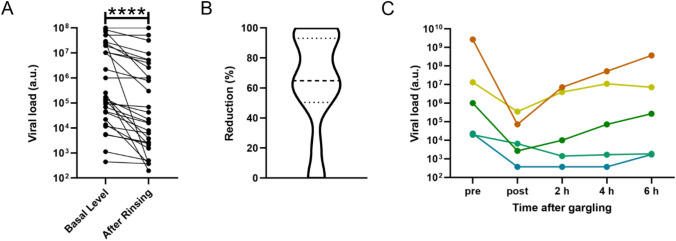
**a** A clear reduction of the viral load was detectable in the population of SARS-CoV-2 patients after gargling with the applied mouth wash solution. This change was statistically highly significant (Wilcoxon matched-pairs signed rank test, with 95% confidence interval, **** ≤ 0.0001). **b** The depiction of the reduction of the viral load shows that it is reduced by a factor of at least two (median: dashed line, quartile: dotted lines). **c** Assay of the time course of the viral load development in the nasopharynx. The viral load requires around 6 h to recover from the rinsing process

We observed a clear reduction of viral load subsequently to the execution of the mouth washing procedure. This reduction was highly significant in the tested population. We determined the relative reduction of viral load at least twofold and the majority of patients exhibited a tenfold reduction of viral load. This was independent of the initial viral load (data not shown). Hence, the patients with high viral titers (above 10^6^ a.u.), which are suspected to be highly infectious, likewise showed an effective reduction in their infectivity. To investigate the temporal development of the pharyngeal viral load we sampled the viral load of five patients at different time points (0 h, 2 h, 4 h and 6 h) after gargling (Fig. 4). We determined that the viral load required approximately six hours to recover to the initial viral load. Interestingly, these results showed that highly infectious patients were able to restore their initial viral load during this time, while less infectious patients were not able to restore their initial infectivity 6 h post gargling.

## Discussion

We could verify that all tested substances (except of substance B) showed a fair in-vitro biocompatibility at concentration smaller than 1% (v/v). At higher concentrations (above 5% v/v) an increase in cellular stress or decrease of viability, respectively, was detected. We assume that the preservatives phenoxyethanol and sodium benzoate reduced the in-vitro biocompatibility of the mouth wash formulations. To test the substances for their immunomodulatory properties, we established cell culture model resembling the immune response to viral infections. We could detect a significant anti-inflammatory potential of the applied test substances E and F. Interestingly, these two substances contained zinc and dexpanthenol as bioactive ingredients, which were both already known for their anti-inflammatory potential [12, 15]. To investigate this effect in greater detail, we designed new formulations resembling these promising candidates E and F but omitting the preservatives and introduced a new substance D only containing dexpanthenol. As suspected, these formulations could be applied at very high concentrations [up to 40% (v/v)] in our cell culture model. Hence, they represented the ideal tool to investigate the immunomodulatory properties of the utilized ingredients zinc and dexpanthenol at concentrations the pharyngeal epithelium will be exposed to during gargling. When these substances were applied at a concentration of only 5% (v/v) to our cell culture model, the amount of dexpanthenol and zinc was already sufficient to dampen the expression of the cytokines TNF-α and IL-6 by a factor of at least 2 up to 7. It is known that these two cytokines are expressed upon activation of the canonical NF-κB pathway which results in the nuclear translocation of the NF-κB transcription factor [10]. In contrast to TNF-α and IL-6 the expression of the chemokines CXCL-9 and CXCL-10 is regulated via the activation of AP-1 thru MAPKs upon TLR-3 ligation [21]. Since these two chemokines were influenced by dexpanthenol alone, we suggest that this inflammatory signaling is only positively regulated by dexpanthenol. Most importunately the antiviral inflammatory response, which is mediated via INF-β, OAS-1 and MX-1 was not negatively influenced neither thru zinc nor by dexpanthenol. Subsequent to TLR-3 activation, the expression of these antiviral proteins is induced by the transcription factor IRF-3 [4]. In Summary, we conclude that zinc might interfere with the canonical pathway of NF-κB, while dexpanthenol additionally modulated MAPK driven activation of AP-1. None of the two bioactive components of our experimental mouth wash formulations seem to interact with the TBK1 driven nuclear translocation of IRF3. Even these novel in-vitro formulations could be applicable to formulation in future clinical studies. Apart from the application of zinc and dexpanthenol in mouth washes one might also consider these two components, which possess very little side effects, as an adjuvant treatment to heavily inflamed lung tissue. This could augment the treatment of COVID-19 patients with, e.g. dexamethasone by further reducing IL-6, TNF-α, CXCL-9 and CXCL-10 expression without interfering with the antiviral signaling so desperately needed in this situation.

This pilot study shows that the mouthwash Linola sept reduces the viral load in the pharynx up to approximately 90%. The reduction in viral load detected by RT-qPCR examined in this study is directly coupled to the infectivity of a COVID-19 patient. La Scola and colleagues found that 70% of samples with Ct values of 25 or below could be cultured, compared with less than 3% of the cases with Ct values above 35 [14]. Since we could observe an average enhancement of the Ct around 3.1 in the observed cohort, we suggest that the gargling reduced the infectivity of the patients significantly. Another study by Singanayagam et al. [26] showed the heaviest reduction of cultured viral particles between a Ct value of 25 and 30; thus we conclude that particularly for patients exhibiting initial Ct values around 25 the treatment with the mouth wash solution is most effective in the reduction of transmission of SARS-CoV-2. It can be assumed that the observed reduction is mainly achieved be pure physical means, thru the hydrodynamic forces acting on the oral epithelium during gargling. But besides that, the formulation of several ingredients in the applied mouth washing solution is designed to augment this mode of action. For example, the component sodium dodecyl sulphate (SDS) acts as an detergence and we suggest that SDS might improve the release of viral particles attached to the oral epithelium into the mouth gargling solution. In terms of the durability of this improvement, we found a correlation between initial Ct value and ability to recover the viral load in the pharyngeal region. We suggest that this faster recovery of SARS-CoV-2 is due to high initial viral titer in combination with the high reproduction rate of SARS-CoV-2 [23] and the high expression of ACE-2 in oral tissue [6]. This viral recovery might be further slowed down by adding active ingredients contained in the applied mouth rinsing solution. For example, the component carrageenan is known to inactivate SARS-CoV-2 [25]. An addition to that, SDS, primarily included due to its detergence properties, was demonstrated to inactivate enveloped viruses like the HIV [30]. Anyhow, a pharmaceutical action needs to be verified against a placebo in further clinical studies. The initial formulation of the applied mouth washing solution Linola sept^®^ not only containing zinc but also hydroxyapatite, also known for its anti-inflammatory potential [32]. In accordance with that the product Biorepair^®^ (test substance A), which is similar to the applied Linola sept^®^ mouth rinse, already displayed a positive immunoregulatory modulation (Fig. 1). We suggest to additionally supplement the rinsing applied in our pilot study with dexpanthenol to further increase the positive effect on the immune system. A long-term observational (above 5 days) placebo-controlled study is currently ongoing to detect an improvement of clinical parameters. A comparison with a placebo group is employed to distinguish the influence of purely mechanical–physical cleaning from the effect of the ingredients. A reduction of viral load in the pharyngeal region in combination with an improved inflammatory response is anticipated.

## Conclusions

This investigation will direct future clinical studies for an improved reduction of viral load in the pharyngeal region. We could determine possible additive applications supporting antiviral therapies already applied in COVID-19. More importantly, we found that the mouth washing can reduce the viral load by 90%. This should lead to a reduced infectivity of the patient infected with SARS‐CoV‐2 and might improve the protection of health care professionals.

## Supplementary Information

Below is the link to the electronic supplementary material.

Supplementary file1 (DOCX 443 kb)
