# Supplementary material for: Mouthrinses against SARS-CoV-2: anti-inflammatory effectivity and a clinical pilot study
Source: Eur Arch Otorhinolaryngol. 2021 May 22;278(12):5059–67. doi: 10.1007/s00405-021-06873-8 (PMC8140561; doi:10.1007/s00405-021-06873-8)

**Fig. S1: Find the right target for optimal concentration of Poly (I:C) in cell culture model to study virus infections.** The stimulation with different concentration of the TLR-3 agonists Poly (I:C) for 4 h was executed on epidermal cells derived from nasopharyngeal tissue. **a** The proinflammatory mediators TNF-α, CXCL-9, CXCL-10 as well as **b** the antiviral targets Interferon Beta, MX-1 and OAS1 showed a robust upregulation at 10 µg/ml.


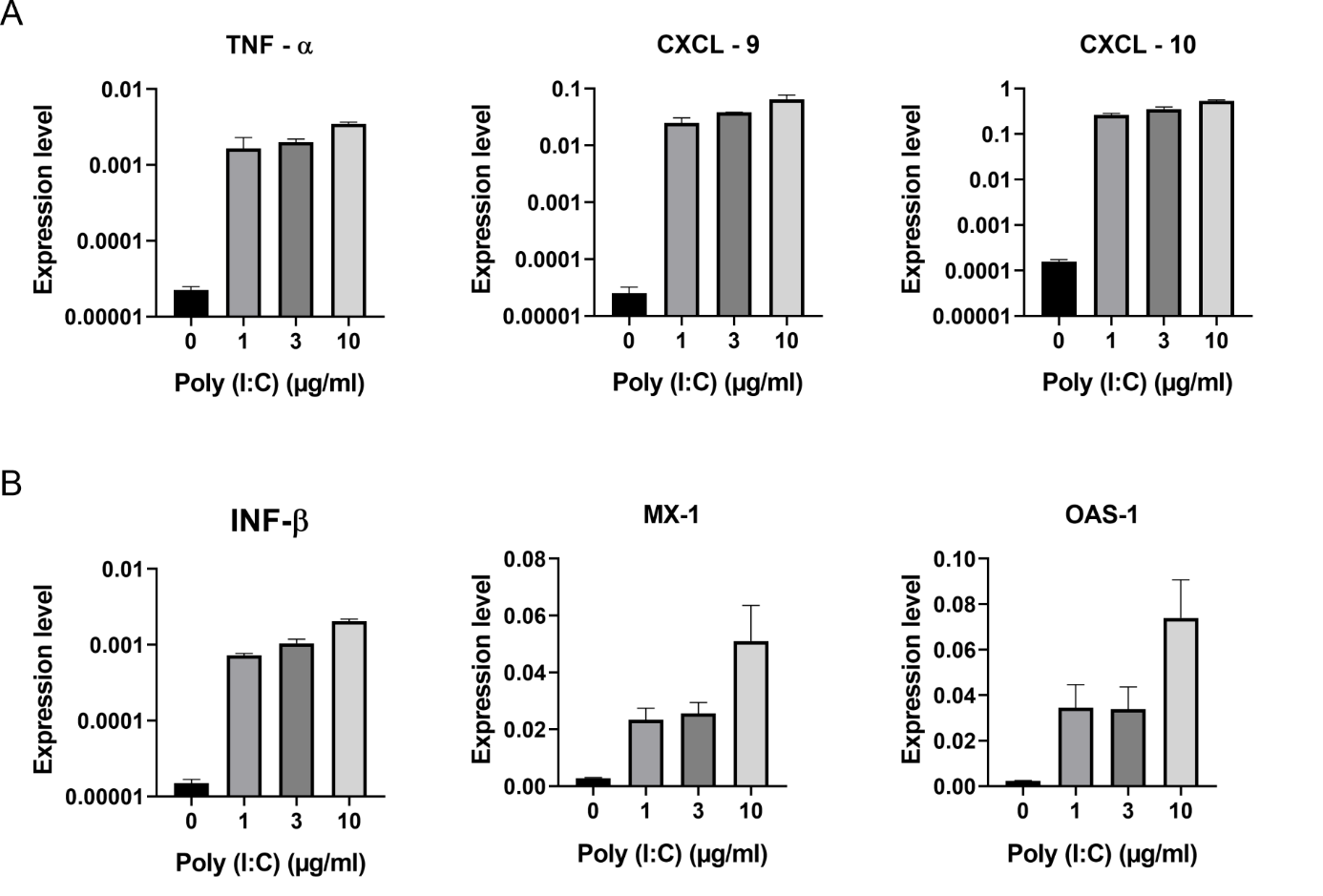


**Fig. S2: 100 times concentrated substances E100 and F100 induce protein coagulation and 10 times concentrated substance E10 and F10 showed same biocompatibility as E and F.** The highly concentrated substances 100 x E and 100 x F precipitated components of the nutrition medium. The less concentrated substances 10x E and 10 x F caused much less of this effect but is still detectable. The vitality changes insignificantly up to a concentration of 1.5 % (v/v) for substance E10 and E and 8 % for substance F and F10. Upon that both showed a nearly parallel descend in viability (To compare the two concentrated substances E10 and F10 with E and F, their applied concentration was divided by ten).


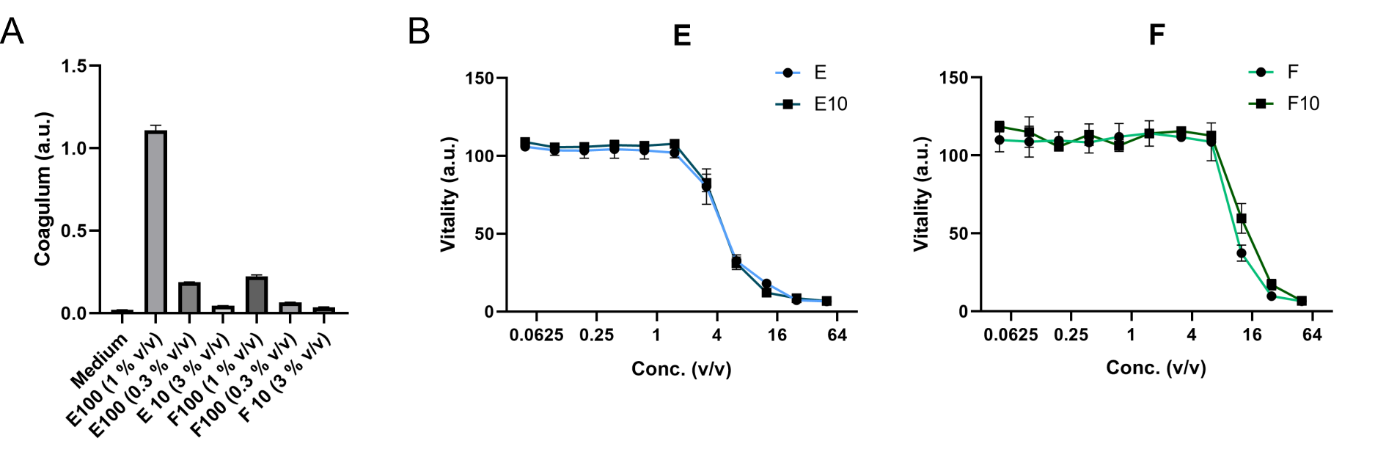

Supplement: Supplementary file 1 — Supplementary file1 (DOCX 443 kb) [file 405_2021_6873_MOESM1_ESM.docx]
